# Sex differences in the ^1^H NMR metabolic profile of serum in cardiovascular risk patients

**DOI:** 10.1038/s41598-019-38881-4

**Published:** 2019-02-20

**Authors:** Ignasi Barba, Mireia Andrés, Irene Picón, Santiago Aguade-Bruix, David Garcia-Dorado

**Affiliations:** 1 Cardiovascular Diseases Research Group, Department of Cardiology, Vall d’Hebron University Hospital and Research Institute, Universitat Autònoma de Barcelona, Barcelona, Spain; 2 Centro de Investigación Biomédica en Red sobre Enfermedades Cardiovasculares (CIBER-CV), Barcelona, Spain

## Abstract

Personalized diagnosis and risk stratification of cardiovascular diseases would allow optimizing therapeutic strategies and lifestyle changes. Metabolomics is a promising technique for personalized diagnosis and prognosis; however, various physiological parameters, including sex, influence the metabolic profile thus hampering its translation to the clinic. Knowledge of the variation in the metabolic profile associated with sex would facilitate metabolomic translation to the clinic. The objective of the present work was to investigate the possible differences in the metabolic ^1^H NMR profile associated to sex beyond lipoproteins. ^1^H NMR spectra from whole serum and methanol deproteinized samples from 39 patients (22 males, 17 females) between 55–70 years old with suspected coronary artery disease that underwent a stress test that was considered negative where included. Deproteinized serum could be used to differentiate sex based on higher levels of lactate and glucose in women. Lipoprotein region was the most variable area of the spectra between individuals, but spectra of whole serum were able to differentiate sex based on lipoproteins. There are sex-related differences in the ^1^H NMR metabolic profile of individuals with suspected cardiovascular disease beyond lipoproteins. These findings may help the translation of metabolomics to the clinic.

## Introduction

Cardiovascular diseases are the number one cause of death globally [http://www.who.int/mediacentre/factsheets/fs317/en/], they are usually slow developing and can take decades to manifest thus preventive interventions are likely to be most effective if applied early, ideally before symptoms appear^1^. Prevention of cardiovascular risk is effective^1,2^ and current guidelines recommend that cardiovascular prevention should be delivered to the general population and at individual level by promoting healthy lifestyle behavior^3^. Individuals at high risk would benefit the most from preventive strategies; however, the identification of those individuals before symptoms appear is challenging. Personalized risk stratification would allow the identification of the individuals at high risk thus giving the opportunity to optimize therapeutic strategies and lifestyle changes for each individual. Also, once clinical symptoms appear, a personalized diagnosis would improve patient treatment.

Myocardial perfusion SPECT studies are widely used for ischemic heart disease screening in populations that have mobility difficulties, as they allow mixed studies of mild physical stress plus simultaneous pharmacological stress. This association is very common in the elderly, patients with traumatological problems, obese adults, and in those situations where adequate tachycardia cannot be guaranteed. To be able to determine with a blood sample which of these patients have a high risk of ischemic heart disease, could improve the patient’s selection and avoid the inadequate use of these tests that are time-consuming and require the use of radioactive tracers.

Metabolomics or the study of the metabolite profile of biofluids show great promise in the area of personalized diagnosis. Because metabolites are the downstream products of gene expression and protein action, they provide a snapshot of a biological phenotype. ^1^H NMR spectroscopy offers the advantages of high reproducibility, being quantitative, and capability to analyze intact biofluids and tissues with no need for sample separation or preparation^4^ which have made it a technique of choice for metabolomics studies.

Previous studies have been able to grade the severity of coronary artery diseases based on ^1^H NMR metabolomics with over 90% specificity^5^ and, in the same work, it is stated that ^1^H NMR based metabolomics would allow widespread population screening and more efficient drug targeting. However, working with a similar population, Kirschenlohr and cols. showed that when confounding variables including sex were taken into account, the results were not as good as previously reported thus limiting the diagnostic power of ^1^H NMR metabolic profiling of serum^6^. Later, a meta-analysis showed that metabolomics could be indeed used for cardiovascular risk prediction^7^ and identified phenylalanine and mono- and polyunsaturated fatty acids as biomarkers for cardiovascular risk after adjusting for confounding factors including sex. However, at this time, metabolomics offers low diagnostic value for coronary artery disease^8^ and has not yet reached clinical application in other pathologies^9^.

Although it has been described that metabolic markers represent the most obvious kind of biomarkers for clinical application, changes caused by disease could be masked by physiological factors including sex, age, and diet^10^. Biological sex markedly impacts cardiac metabolism at rest^11^ and in response to metabolic diseases^12,13^. Also, lipoprotein profiles, a risk factor for the development of cardiovascular diseases, are also affected by sex^6,14^. Thus, guidelines recommended that parameters known to be associated with cardiometabolic disease, including sex, should be taken into account in metabolomic studies^15^.

There is a need for a population screening tool for individualized risk stratification and diagnosis of cardiovascular diseases once symptoms appear. A first step towards reaching this goal would be to understand the background sources of variation in the metabolic profile of the population most likely to suffer cardiovascular diseases The objective of the present work was to evaluate possible differences associated with sex in the ^1^H NMR metabolic profile of patients with suspected CAD beyond lipoproteins.

## Results

### Patients

Table 1 shows the relevant epidemiological and clinical data of the patients included in the study. Men were significantly taller than women while they had similar weight; as a result, BMI was higher in women than men. Women had higher total cholesterol levels than men; however when lipoprotein subclasses were analyzed individually, differences did not reach statistical significance. All other parameters analyzed, including the incidence of diabetes and pre-test medication were similar between the groups.

**Table 1:** Epidemiological and clinical data of the patients included in the study.

Gender	Female	Male	p
Age	65.5 ± 5.3	66.6 ± 6.2	0.553
Weight (Kg)	75.5 ± 12.9	77.5 ± 9.9	0.582
Height (m)	1.60 ± 0.08	1.69 ± 0.07	<0.001
IMC	29.3 ± 3.6	27.1 ± 3.0	0.035
HTA	13/17	12/22	0.157
Total Cholesterol (mg/dL)	202.8 ± 45.7	171.8 ± 40.7	0.029
LDL (mg/dL)	120.0 ± 31.1	103.6 ± 41.1	0.176
HDL (mg/dL)	54.0 ± 16.2	47.1 ± 7.9	0.095
TAG (mg/dL)	153.6 ± 106.7	115.0 ± 47.0	0.141
Diabetics	5/17 (29.4%)	9/22 (40.9%)	0.550
Glomerular filtration Rate (ml/min/1.73 m^2^)	80.1 ± 12.6	73.2 ± 24.2	0.293
AAS	7/17 (41.2%)	11/22 (50.0%)	0.584
Statins	9/17 (52.9%)	13/22 (59.1%)	0.701
Fibrates	0	0	
Ezetimibe	0	0	
Oral Antidiabetics	4/17 (23.5%)	6/22 (27.3%)	0.791
Anticoagulants	3/17 (17.6%)	2/22 (9.1%)	0.428
Insulin	1/17 (5.9%)	5/22 (22.7%)	0.148

Data are expressed as average standard deviation or number of cases (%) and obtained from the blood test date closer to the stress test.

### Spectra

**Figure 1:**
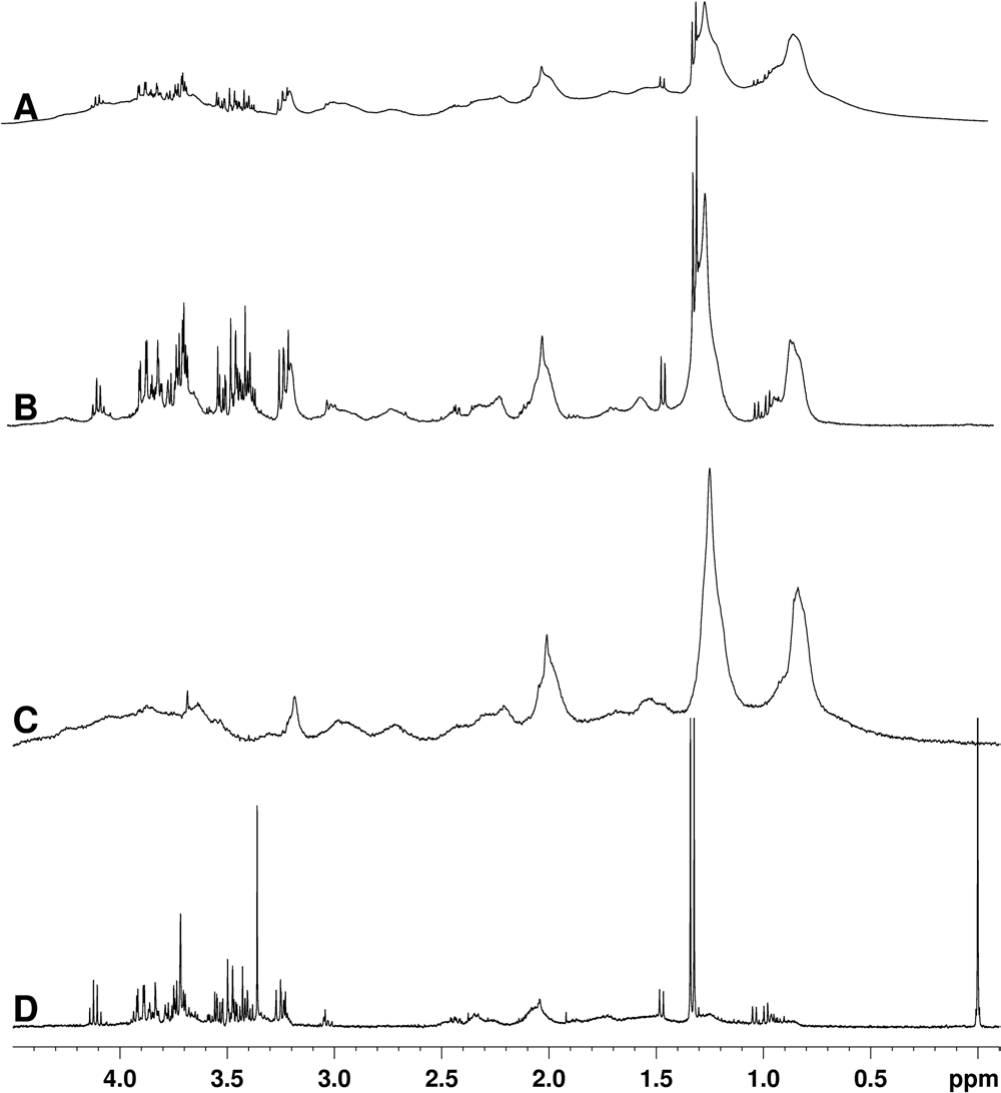
Typical spectra from serum samples. (**A**) Pulse-and-acquire spectra (**B**) CPMG with an effective T2 delay of 32 ms (**C**) Diffusion edited spectra and (**D**) NOESYPR1D spectra obtained from a deproteinized serum sample.

NMR spectra of serum samples obtained in this work were similar to our previously published data^16^. CPMG spectra removed part of the signal originating from lipoproteins thus allowing easier observation of small molecular weight compounds that would otherwise be masked by the large and broad peaks of macromolecules; conversely, diffusion edited spectra removes the signal originating from small molecules (Fig. 1).

Spectra of deproteinized samples (Fig. 1) were similar to those published previously^17^. However, in some spectra traces of the methanol used for precipitation were observed even after freeze-drying the samples; the area of the spectra at around 3.34 ppm, containing the residual methanol peak, was removed from the analysis.

### Pattern Recognition

**Figure 2:**
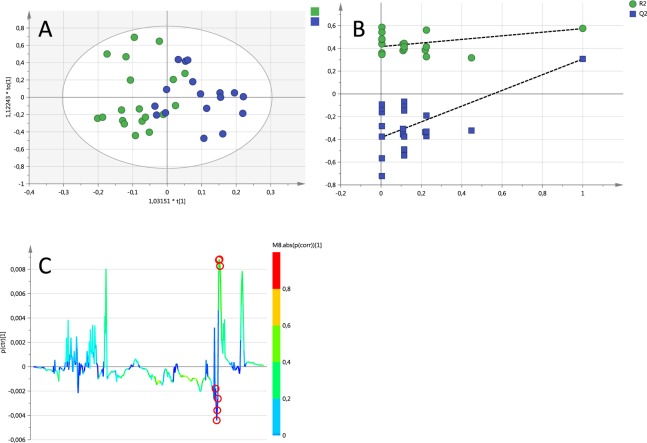
Pattern recognition results from the CPMG spectra (**A**) corresponds to the score plot of the OPLS-DA model, each point corresponds to a sample; green female, blue male. (**B**) Permutation analysis showing the validity of the model and (**C**) S-plot with the 10 most important variables in the discriminant function marked with red circles; all correspond to the methylene lipid peaks.

It is possible to obtain an OPLS-DA (Orthogonal projection to latent structures-Discriminant Analysis) model to differentiate between men and women using spectra obtained with the CPMG (Carr-Purcell-Meiboom-Gill) pulse sequence (Fig. 2A). The model (R^2^x = 0.7; R^2^y = 0.54; Q^2^ = 0.307) is able to correctly classify 80% of the samples. Although permutation tests show that the model is robust (Fig. 2B), CV-ANOVA does not reach statistical significance (p = 0.078) possibly due to the high variation seen in the lipid region between the samples, even in T2 edited spectra.

The variables responsible for the differences between sexes in the discriminant model obtained using T2 edited spectra (Fig. 2A) were found around 1.28 ppm, which correspond mostly to the methylene peak of lipid acyl chains (Fig. 2C).

It was possible to obtain a model able to differentiate sex using diffusion edited spectra (R^2^x = 0.59; R^2^y = 0.25; Q^2^ = 0.07). However, this model is able to correctly classify 72% of the samples but does not reach statistical significance in CV-ANOVA (p = 0.65).

**Figure 3:**
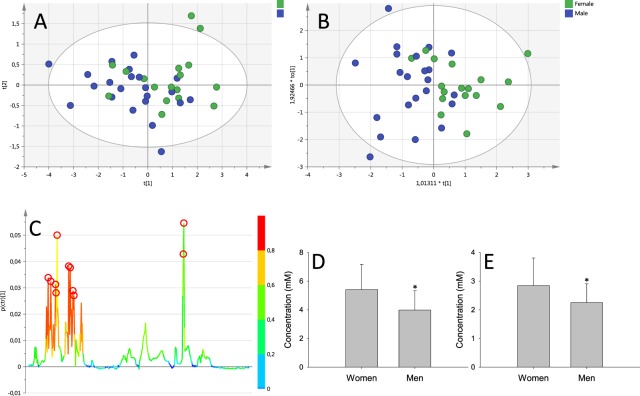
Results of the analysis performed on deproteinized samples. (**A**) First and second components of the Principal component analysis (**B**) OPLS-DA model obtained with the spectra from the deproteinized samples. (**C**) S-plot with the most relevant variables in the discriminant function marked with red circles, they correspond to glucose and lactate. (**D**,**E**) are bar graphs representing the concentrations (in mM) of glucose and lactate in women and men obtained from the spectra of deproteinized serum samples.

Spectra from deproteinized samples show less inter-individual variation than spectra from complete serum. Principal component analysis performed on the spectra showed a tendency towards clustering in the first component suggesting that the main source of variation within the deproteinized dataset is sex (Fig. 3A). It was possible to obtain a statistically significant OPLS-DA model (R^2^x = 0.716; R^2^y = 0.43; Q^2^ = 0.25) (p = 0.039) that was able to classify the spectra according to sex with 80% accuracy (31/39) (Fig. 3B). The variables responsible for the discrimination were found at around 1.35 and 3.70 ppm tentatively assigned to lactate and glucose respectively (Fig. 3C).

We did combine CPMG and deproteinized spectra in a single analysis. The statistical model obtained slightly improves the one obtained using spectra from deproteinized samples (R^2^x = 0.564; R^2^y = 0.511; Q^2^ = 0.302) (p = 0.021) reaching 89% accuracy (35/39) in the classification. However, the most important variables in the discrimination arise from the deproteinized spectra.

Finally, using Chenomx software we were able to identify and quantify 19 metabolites from the deproteinized spectra (Table 2). As expected from the whole spectra analysis, lactate, and glucose where elevated in women. Also, valine and glycine were elevated in women (Table 2); the other metabolites remained unchanged. Metabolite concentrations are in similar ranges to previously published data^18^ [http://www.serummetabolome.ca/].

**Table 2:** Metabolite concentration derived from deproteinized spectra. Data in mmol/L.

	Females	Males	p
3-Hydroxybutyrate	0.202 ± 0.068	0.226 ± 0.084	0.604
Acetate	0.056 ± 0.026	0.058 ± 0.018	0.867
Alanine	0.387 ± 0.148	0.323 ± 0.104	0.143
Betaine	0.038 ± 0.027	0.051 ± 0.040	0.270
Carnitine	0.024 ± 0.017	0.021 ± 0.016	0.610
Choline	0.029 ± 0.010	0.034 ± 0.017	0.265
Creatine	0.053 ± 0.025	0.040 ± 0.018	0.068
Creatinine	0.087 ± 0.021	0.107 ± 0.093	0.385
Glucose	5.408 ± 1.748	3.981 ± 1.343	0.009
Glutamate	0.592 ± 0.247	0.492 ± 0.176	0.169
Glutamine	0.576 ± 0.200	0.493 ± 0.136	0.151
Glycine	0.230 ± 0.092	0.151 ± 0.046	0.002
Isoleucine	0.082 ± 0.027	0.078 ± 0.021	0.601
Lactate	2.843 ± 0.963	2.250 ± 0.627	0.037
Leucine	0.166 ± 0.057	0.150 ± 0.051	0.375
Acetone	0.021 ± 0.009	0.020 ± 0.009	0.815
Threonine	0.190 ± 0.097	0.150 ± 0.064	0.160
Valine	0.261 ± 0.069	0.206 ± 0.051	0.009
sn-Glycero-3-phosphocholine	0.057 ± 0.035	0.050 ± 0.031	0.551

## Discussion

Data presented in this work shows that there are sex-related differences in the ^1^H NMR metabolic profile of individuals with suspected cardiovascular disease beyond lipoproteins.

Using NMR spectra of complete serum we have been able to show that women have higher lipoprotein levels than men. This is consistent with findings in the general population^19^ and could be also seen in a group of patients with cardiovascular diseases^6^. Moreover, in our study, women had higher total cholesterol levels than men, in accordance with general population findings^20^.

We have previously shown that T2 edited spectra of complete serum provide the best results to predict exercise-induced ischemia^16^. In the present work, the discriminant model to differentiate between men and women based on T2 edited spectra was based on lipoproteins as shown before^6^. However, this model did not reach statistical significance when evaluated using CV-ANOVA.

Diffusion edited spectra show only the resonances corresponding to macromolecules, mainly lipoproteins. Sex is known to be associated with blood lipoproteins^6^ suggesting that diffusion weighted spectra would be suitable for differentiating men from women. However, we could not obtain a valid statistical model able to differentiate between men and women; most likely due to the high variability in lipoprotein composition between individuals.

On the other hand, when using deproteinized samples, it was possible to obtain a statistical model able to differentiate men from women mostly based on the higher levels of glucose and lactate seen in women. In our database 14 out of 39 individuals were considered diabetics (were prescribed oral antidiabetics, insulin or both) at the time of analysis but only one individual (female) showed blood glucose over 6.1 mmol/L (limit to be diagnosed as diabetic) on the sample analyzed. In a study involving healthy human volunteers, women had shown higher glucose levels in urine but not in plasma^19^ while in another healthy population of young adults men showed higher glycaemia than women^21^. Metabolite quantification of deproteinized serum samples showed increases in glucose, lactate and also of valine and glycine in women. Glycine has been found elevated in younger^21^ and older^22^ healthy women. On the other hand, valine was found to be higher in healthy older men than women^22^. However, we could find no reports based on individuals with suspected cardiovascular diseases as the ones described in this study.

There are some reports in the literature describing differences in the metabolic profile associated with sex^19,23–25^. However, this is the first one focusing on the population with suspected cardiovascular diseases, which would benefit most from a non-invasive screening and diagnostic tool.

### Study limitations

We are aware that the number of patients included in this study is limited; however, in well defined populations we^16^ and others^26^ have been able to obtain positive results for serum metabolomics in CAD patients. In order to minimize the effect of a limited sample size, we evaluated PLS-DA models using CV-ANOVA a robust approach and dependent on sample number. The percentage of diabetics in our study is similar to what is found in primary care for similar age groups in our environment with with slightly higher incidence of men than women for similar age groups^27^. Diabetes is known to influence the metabolic signature however, when patients with glycated hemoglobin higher than 6 were removed from the analysis it was still possible to obtain a statistical model able to differentiate sex (CV-ANOVA p = 0.029) and there was no change regarding the variables responsible for group discrimination. However, further studies with larger populations should be done in order to validate our work.

In conclusion, we have detected differences in the ^1^H NMR metabolic profile between men and women in a population with suspected cardiovascular disease in deproteinized serum samples. These findings may facilitate the development of ^1^H NMR based metabolomics approaches in cardiovascular diseases and its translation to the clinic.

## Methods

### Patients

39 consecutive patients (22 males, 17 females) between 55–70 years old that were referred to perform myocardial perfusion SPECT study with stress test at Hospital Vall d’Hebron were included in this study. Patient selection was done prospectively but only the samples of those studies considered negative in the report were taken, considering negative if the clinical stress test, the ECG, the gammagraphic images and the ventricular function were all normal. Patients that were unable to perform a full stress test or required pharmacological stimulation where excluded from the study.

Patients included are from the METS (Metabolomic Profile of Patients Undergoing Myocardial Perfusion SPECT study (ClinicalTrials.gov Identifier: NCT02968771). All patients gave their written informed consent and the study was approved by the “Hospital Vall d’Hebron” ethics committee. Methods and procedures were performed in accordance with local guidelines and regulations.

### Samples

Blood samples (5 ml) were obtained just before the stress test after overnight fasting. Blood was allowed to clot at room temperature, then, centrifuged at 1000 g, 4 °C, 5 minutes. The serum was separated and kept at −80 °C until needed.

### Serum deproteinization

Serum was deproteinized using methanol precipitation^17^. Briefly, 200 μl of serum were mixed with 400 μl of methanol, vortexed and kept at −20 °C for 20 minutes. Afterward, the sample was centrifuged at 11000 g, 4 °C, 30 minutes. The supernatant was lyophilized and stored at −80 °C until NMR analysis.

### NMR spectroscopy

Prior to NMR spectroscopy, deproteinized samples were reconstituted in 600 μl of PBS (Phosphate Buffered Saline) made up with D_2_O, containing 0.5 mM TSP (Trimethylsilyl tetradeuteropropionic acid sodium salt) as a concentration and chemical shift reference and placed in a 5 mm NMR tube. Spectra were acquired at 300 K on a 400 MHz vertical bore magnet interfaced to a Bruker Avance console. Each spectrum consisted in the accumulation of 64 scans with a NOESYPR1D (One-dimensional Nuclear Overhauser Spectroscopy) pulse sequence with a mixing time of 100 ms.

Serum samples (200 μl) were mixed with PBS-D_2_O (300 μl) just prior to NMR spectroscopy. A series of spectra including pulse-and-acquire, CPMG with an effective T2 delay of 32 ms and diffusion edited spectra were acquired for each sample.

### Data analysis

For pattern recognition, each spectrum was manually phase corrected and the area between 0.5 and 9 ppm (excluding the water zone) divided into bins of equal width of 0.01 ppm. The resulting digitized spectra were normalized to total area of 1 and fed into SIMCA v14 software (Umetrics, Umea, Sweeden) for further processing. Pareto scaling was applied to the data.

General variance within the dataset was analyzed using principal component analysis (PCA)^28,29^. This method reduces the dimensionality of a data set while retaining as much as possible of the variation present in the original data set facilitating the extraction of information. Being an “unsupervised” approach, it does not require input from the observer and, thus, is free from possible bias.

In order to assess the capacity of the NMR spectra to differentiate between samples from men and women, a supervised classification analysis was performed. Supervised classification refers to the development of a statistical model able to differentiate two (or more) populations defined in advance. The target is to assign an individual to one of the populations. The information for this classification is provided by a “training set” of correctly classified individuals. In our case the ability of the classification models was tested using the “leave-one-out” approach where one in seven samples within the dataset were not used to define the model and then used to test how well the classification algorithm worked; this process is repeated in an iterative process until all the samples have gone through being in the training and test sets. The supervised approach used in this work was Orthogonal PLS discriminant analysis (OPLS-DA) that highlights the variables responsible for differences among classes^30^. All OPLS-DA models were able to classify the samples better than random grouping but were only considered statistically significant when CV-ANOVA^31^ was <0.05.

Metabolite quantification was performed in deproteinized spectra using Chenomx software (Chenomx, Edmonton, Canada) by comparing the areas of the peaks of interest to that of TSP added as an internal standard at a final concentration of 0.5 mM. Metabolite concentration is given in mmol/L ± standard deviation and concentrations were compared using unpaired, two-sided t-test without correction for multiple comparisons.

## Data Availability

The datasets generated during the current study are available from the corresponding author on reasonable request.
